# A Rare Case of Terminal Ileum Gastrointestinal Stromal Tumor in a Young Caucasian Adult

**DOI:** 10.7759/cureus.51340

**Published:** 2023-12-30

**Authors:** Abhinav K Rao, Theshali Anthony, James Ravenel, Joanna Kosko, Ian Whitaker

**Affiliations:** 1 Internal Medicine, Trident Medical Center, North Charleston, USA; 2 Radiology, Charleston Imaging Specialists, Charleston, USA; 3 Pathology, Trident Medical Center, North Charleston, USA

**Keywords:** gastrointestinal neoplasms, gastrointestinal bleeding, terminal ileum, abdominal imaging, gastrointestinal stromal tumor (gist)

## Abstract

Gastrointestinal stromal tumors (GISTs) are rare in young individuals and typically affect older adults. We present the case of a previously healthy male who presented with severe hematochezia, fatigue, and dizziness. Colonoscopy did not demonstrate any colonic mass. CT of the pelvis with contrast revealed a pelvic mass measuring 7.4 cm. Biopsy confirmed a low-grade mixed-type GIST of the terminal ileum. Surgical resection was successfully performed. Histopathological analysis further characterized the tumor, and the patient was discharged with consideration of adjuvant imatinib therapy. This case underscores the importance of thorough diagnostic evaluation and multidisciplinary management for atypical presentations of gastrointestinal bleeding in young patients.

## Introduction

Gastrointestinal stromal tumors (GISTs) are a rare type of mesenchymal neoplasm, compromising 1-2% of primary gastrointestinal (GI) cancers [1]. They are most common in older adults, with a median diagnosis age of 65-69 years, and much rarer in those younger than 20 years. Furthermore, among all cases of GIST, only 2% occur in children and young adults [2]. Among young adults, there tend to be underlying genetic predispositions, often linked to germline KIT and PDGFR mutations, which are the most commonly associated mutations with GIST [3,4]. GISTs are not gender-specific but are more prevalent in African Americans [5]. GIST may present with abdominal pain, bowel obstruction, nausea, vomiting, and GI bleeding. Common sites for GISTs include the stomach and jejunum/ileum, while they are less frequently found in the esophagus, duodenum, colorectum, and anus [6]. The differential diagnosis is broad and should include any cause of GI bleeding, gastric stromal tumors, or other malignancies such as gastric adenocarcinoma, lymphoma, and carcinoid tumors [7]. In this report, we present a rare case of a primary GIST located in the terminal ileum in a young Caucasian male.

## Case presentation

A 21-year-old Caucasian male with no significant past medical history presented to the emergency department with severe hematochezia that started one week ago and was accompanied by fatigue and dizziness. He underwent a colonoscopy two months prior to presentation due to similar symptoms, which failed to demonstrate any abnormal findings. On presentation, he denied any abdominal pain, fevers, chills, night sweats, or weight loss. He did report some remote and intermittent bloody bowel movements over the last three months and had reportedly undergone a colonoscopy three months ago that was negative. His family history was significant for pancreatic and prostate cancer in the maternal grandparents. He reported drinking socially and denied any history of smoking or recreational drug use.

**Figure 1:**
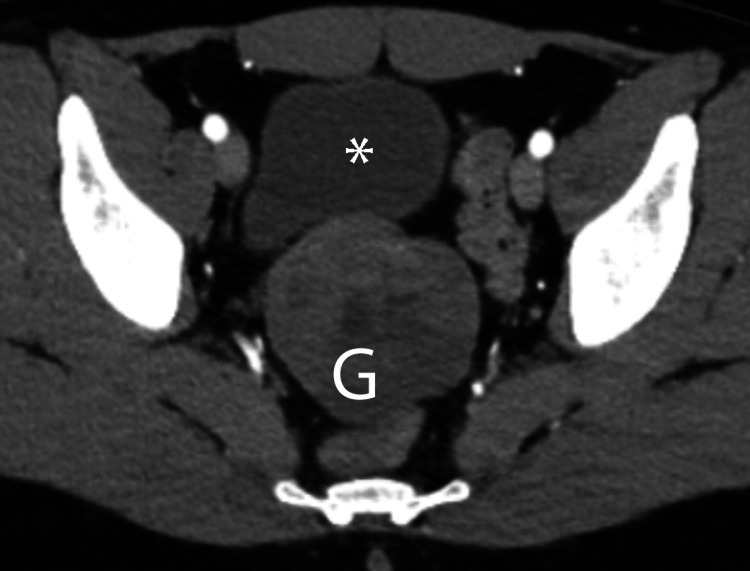
Ileal gastrointestinal stromal tumor Axial contrast-enhanced arterial phase image reveals heterogeneous ileal mass (G) just posterior to the urinary bladder (*).

**Figure 2:**
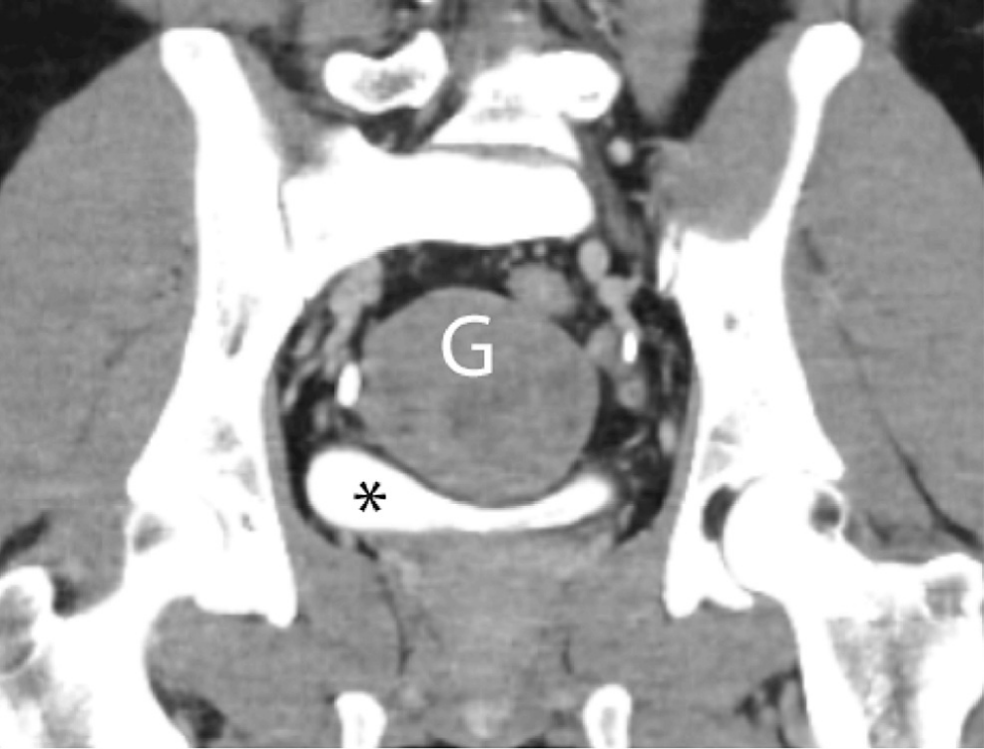
Ileal gastrointestinal stromal tumor. Coronal contrast-enhanced delayed phase reveals the relationship of the mass (G) with the urinary bladder (*).

**Figure 3:**
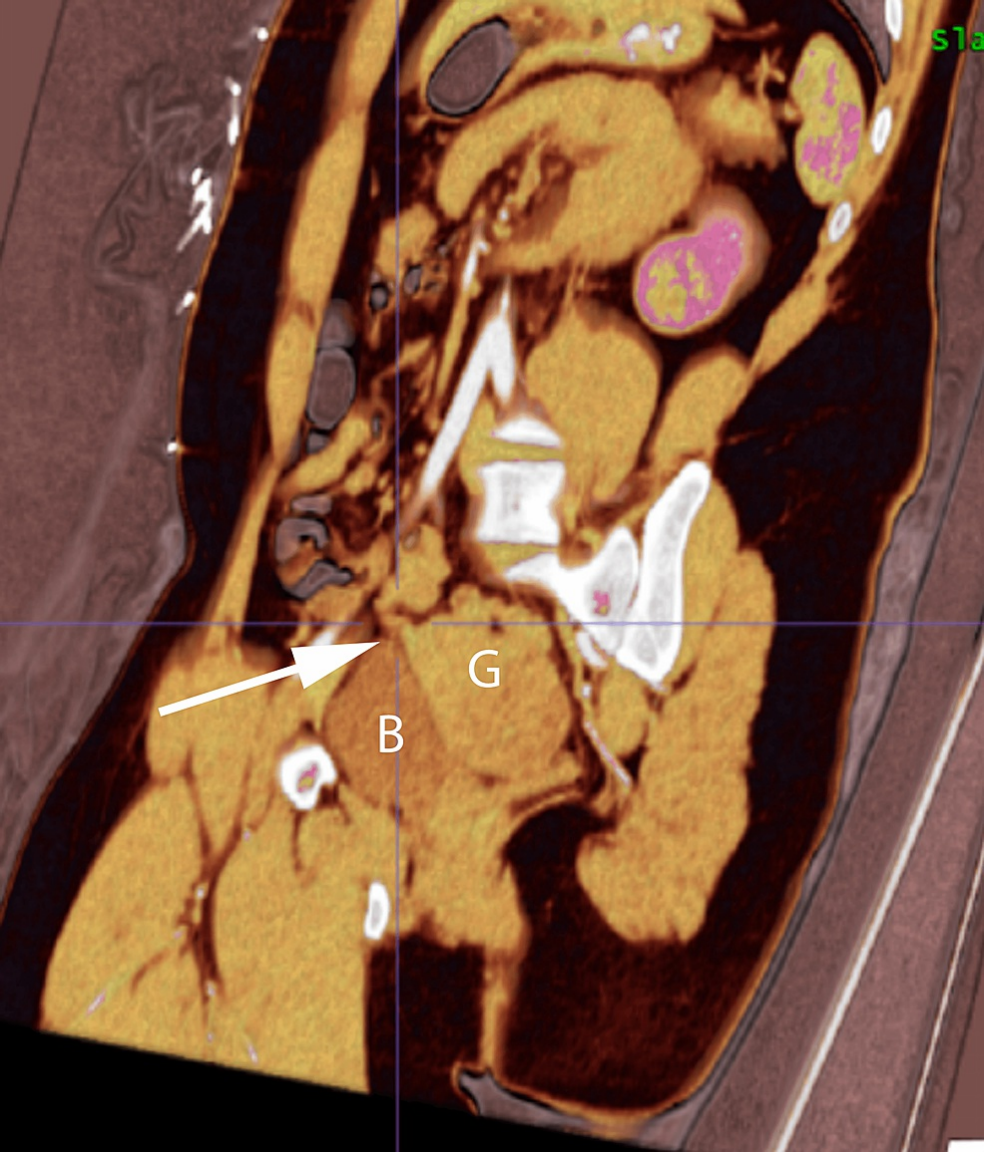
Gastrointestinal stromal tumor extending from the terminal ileum. Gastrointestinal stromal tumor (G) measuring 7.5 cm in the midline lower pelvis located anterior to the rectosigmoid region and posterior to the bladder (B). This soft tissue mass appeared to be extending from the terminal ileum. There is also mild wall thickening of the ascending colon and proximal transverse colon consistent with colitis (not shown in provided images).

On presentation to the emergency department, he was tachycardic to 113 with otherwise normal vital signs. His labs were significant for a hemoglobin of 6.4 and a mean corpuscular volume of 70. Computed tomography angiography (CTA) of the abdomen and pelvis was performed, which was negative for evidence of an active GI bleed but did demonstrate mild wall thickening of the ascending colon and proximal transverse colon, consistent with inflammatory colitis, and a 7.5 cm soft tissue mass in the midline lower pelvis located anterior to the rectosigmoid region and posterior to the bladder. This mass appeared to be extending from the terminal ileum (Figures 1-3). Given the appearance of the mass, the differential considerations included GIST, lymphoma, and sarcoma. The vascular supply appeared to be from the superior mesenteric artery and vein. He underwent computed tomography (CT) guided biopsy of the mass, and specimens were sent for pathology. Due to ongoing bleeding despite a negative CT, a tagged red blood cell (RBC) scan was performed, which revealed active GI hemorrhage within the mid-abdomen at the level of the aortic bifurcation. Capsule endoscopy was performed, which corroborated hemorrhage. Colonoscopy revealed a polyp at the ileocecal valve with resolved bleeding, for which biopsy was taken, and also revealed lymphocytic infiltration without other abnormalities.

On day 6 of his hospitalization, his pelvic mass biopsy results came back positive for GIST, low grade, mixed type, of the terminal ileum. CT of the chest was performed, which did not reveal evidence of metastatic disease. Surgical resection was performed via laparotomy with segmental small bowel resection, reanastomosis, and incidental appendectomy. He was transfused throughout the hospital course, requiring 7 units of packed RBCs, to maintain a hemoglobin of greater than 7.

**Figure 4:**
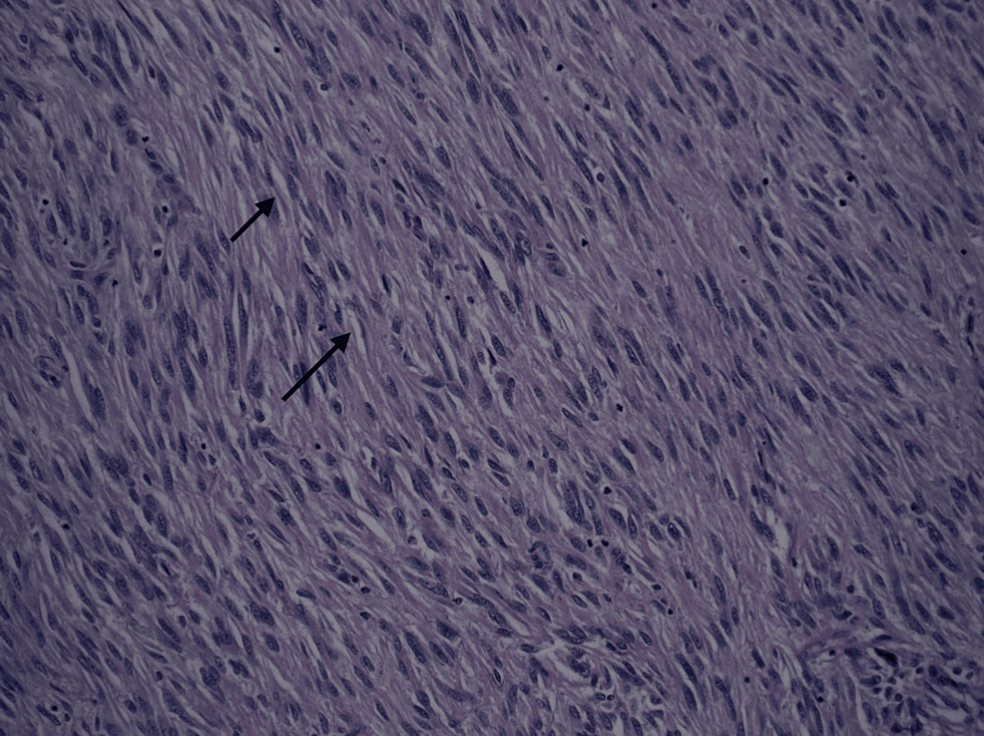
Spindled cells with occasional perinuclear vacuolization consistent with gastrointestinal stromal tumor. H&E stain (200X magnification).

**Figure 5:**
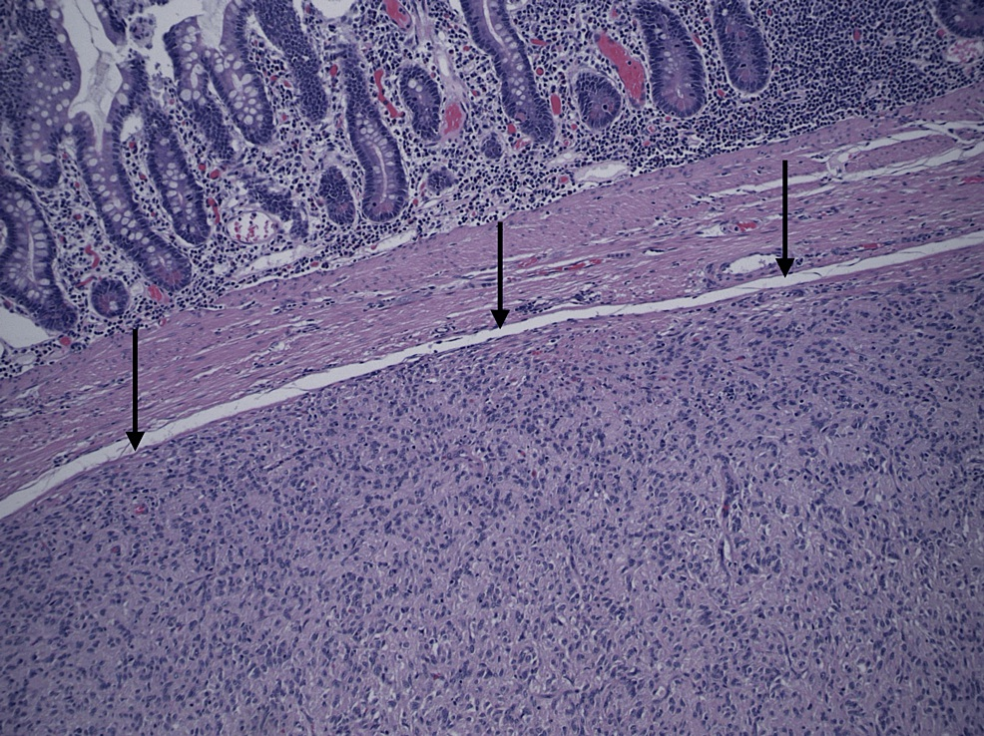
Submucosal small bowel mass composed mainly of spindled cells with focal epithelioid cells, consistent with mixed type gastrointestinal stromal tumor, grade 1 (low grade). H&E stain (100X magnification).

**Figure 6:**
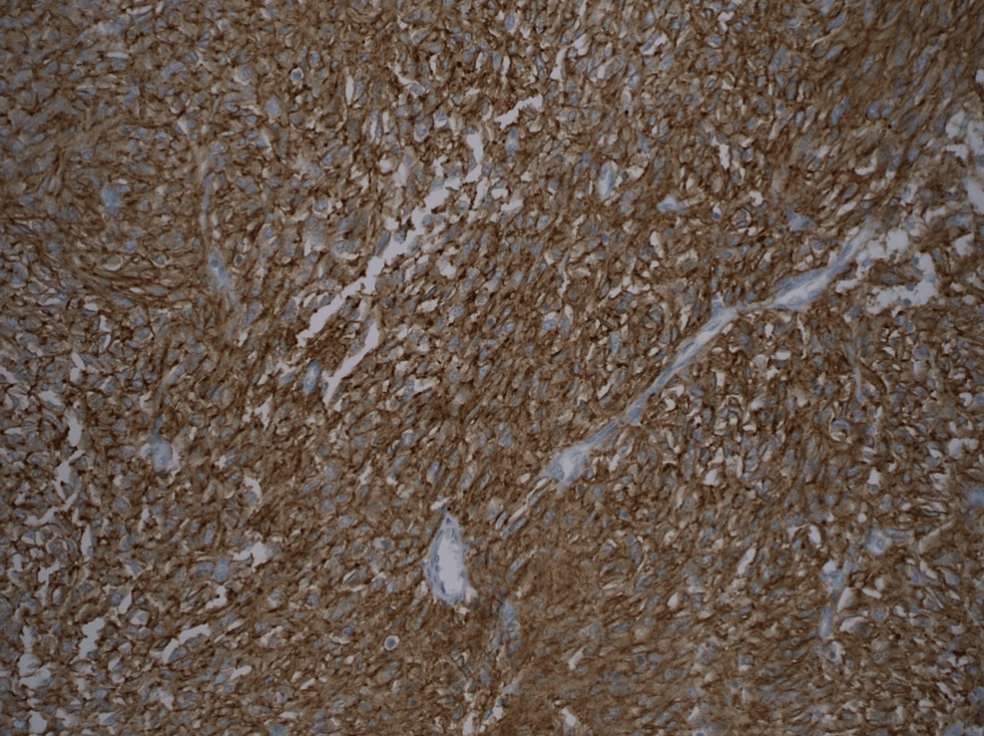
Gastrointestinal stromal tumor with diffuse positive staining for DOG-1 in tumor cells. DOG-1 immunohistochemical stain (200X magnification).

Histopathological evaluation revealed a 7.3-cm low-grade GIST of the terminal ileum, predominantly spindle type (70%) with a low mitotic rate (1 per 5mm²), no necrosis, clear margins, and a low K1=67 proliferation index (1-2%). Immunohistochemistry showed positive staining for CD117 and DOG-1, while cytokeratin AE1/AE3, CD34, S-100, and SMA were negative (Figures 4-6). The patient was followed by gastroenterology, oncology, and general surgery throughout his hospital course. He was discharged from the hospital with consideration of outpatient adjuvant imatinib therapy. Mutations for *KIT*, *PDGFR*, and *SDH* were sent by his outpatient oncologist, which returned negative. He continues to follow up with oncology and is doing well, with no reemergence of his cancer on serial imaging at six months.

## Discussion

In this case, this young patient was being investigated for upper GI bleeding when incidentally he was found to have a radiological finding of a pelvic mass. The mass discovered was so large that the organ of origin was somewhat unclear; however, it appeared to be extending from the terminal ileum. The CTA study provided us with a rapid and reproducible assessment of the size of the tumor as well as its relationship with other structures and confirmed the lack of metastatic disease.

In this clinical presentation with a fairly moderate tumor size and symptomatic GI bleeding, the mainstay of treatment is surgical excision with negative margins. According to the Armed Forces Institute of Pathology prognostic model, this patient had a intermediate or moderate risk of disease progression (24%) during long-term follow-up when considering tumor size (greater than 5 cm but less than 10 cm), mitotic rate (≤5/10 high power field (HPF), and location in the ileum [8]. Traditional chemotherapy and radiotherapy have not been effective in GIST, and the current consensus is obtaining molecular assessments to guide the use of systemic tyrosine kinase inhibitors such as imatinib. Molecular targeted therapy agents, a fairly recent advancement only initiated in 2000, work by competing for the ATP binding site on the target kinase, inhibiting tyrosine kinase and reducing cellular proliferation. Imatinib, with a standard duration of three years, gained FDA approval in 2008 for preventing recurrences in operated GIST among both intermediate- and high-risk groups [9]. Despite the moderate risk of relapse, which is a typical indication for adjuvant imatinib, in this case, we achieved complete tumor resection and opted for close follow-up with serial CT imaging to monitor for recurrence.

This patient’s case underscores the importance of comprehensive diagnostic evaluation including advanced imaging and histopathological analysis in the successful diagnosis and management of a rare GIST in an atypical age group and location. The collaborative efforts of various medical specialties and timely surgical intervention along with targeted adjuvant therapy ensured a favorable outcome for this young patient. On follow-up, it was noted that a first-degree relative was having GI symptoms as well, which needed to be further investigated.

## Conclusions

We presented here a rare cause of terminal ileum GIST in a young Caucasian adult. Timely and appropriate treatment resulted in complete eradication of his tumor, with no recurrence seen on serial imaging at six months. As there were no discernible genetic mutations and an absence of high-risk indicators, elective molecular therapy was not pursued. This case underscores the importance for physicians to recognize the potential for GIST in young adults, particularly as an uncommon source of GI bleeding.
